# The Geopolitics of Expert Knowledge: Analysing the European Commission’s Role and Influence in Indonesian Higher Education

**DOI:** 10.1057/s41307-025-00429-9

**Published:** 2025-11-25

**Authors:** Sotiria Grek, Hilary MacDonogh

**Affiliations:** 1 University of Glasgow, Glasgow, UK; 2 University of Edinburgh, Edinburgh, UK

**Keywords:** Expertise, Infrastructure, Quality, Universities, Geopolitics, Indonesia

## Abstract

Building on a rich set of policy documents and interview data, and using the concept of the epistemic infrastructure, the article focuses on an analysis of how European HE expert actors became the preferred advisers for Indonesia’s HE quality assurance system. As the article shows, working towards the quality assurance of higher education institutions in Indonesia serves not only for the apparent purpose of offering technocratic and rational solutions to improving HE governance and delivery, but also for the parallel aim of expanding the role and influence of European higher education in other world regions and zones of influence. Given the role and identity of the EU as a political project, as well as the specific mission that has been assigned to European universities as custodians and carriers of European liberal values, the work of European experts in Indonesia has geopolitical value and significance; it thus allows us to examine the geopolitics of expert knowledge production as a new and complex space of interactions, possibilities and contradictions that calls for a nuanced and careful analytical approach.

## Introduction

Although most elite universities across the world have always prided in being outward-looking and interconnected, the internationalisation of higher education has become a particularly dominant phenomenon over the last 50 years. Universities seek to attract global talent, not only in their student cohorts and workforce, but also in the expert knowledge that guides their decision-making to best be able to cope with a highly volatile world. Although in the second half of the twentieth-century Indonesia’s higher education had primarily been within the World Bank’s (WB) zone of expert influence, in recent years there has been a significant shift: the European Commission’s DG EAC has taken an active role in becoming an expert policy actor in higher education in the country. Analysing interview data with expert actors and policymakers both in Europe and in Indonesia, this article traces how the Indonesian weaning off from the WB led to the preference for DG EAC as the expert actor for collaboration. Indonesian policy actors appear to choose to work more closely with the Commission’s expert knowledge, especially in relation to quality assurance policies and practices, as tried and tested through the process and the rise of European HE quality assurance infrastructure. Thus, during the last decade, the European Union, represented not only by the European Commission, but also the numerous European quality assurance agencies, has come to be key intergovernmental organisation^1^ (IO) that supports Indonesian higher education policy making.

As this article will show, this has been a strategic, geopolitical shift that has long been in the making: on the one hand, the European Commission has always aspired for European universities to represent European values and the distinctive “European way of life” (von der Leyen, 2019) globally; on the other, Indonesia has searched to break from leaning on WB loans, while continuing to internationalise and learn from abroad. The article will show how Indonesian experts and policy makers discuss the problematic nature of the interdependency with the WB, versus the more flat and open method of work of European experts, not in many ways dissimilar from the principles and soft governing tools of the Open Method of Coordination.

This article foregrounds three key arguments in the analysis of Indonesia’s shift in their expert actor of choice. Firstly, as we will show, the political work of “soft expertise” became the main vehicle for this emergent alliance: by soft expertise, we refer to the incremental epistemic infrastructuring of expert knowledge in quality assurance in Europe, a process that allowed the creation of a “bounded” harmonisation, aligned with the long history of governing education in Europe: by setting broadly defined standards and values, it leaves plenty of room for policy divergence and decision-making manoeuvre for the highly varied and nationally defined higher education systems of Europe. If the WB was the more inflexible and restrictive expert IO, Indonesia’s higher education experts found in the European quality assurance system a way to align with international standards of high quality, while still pursuing HE as a sovereign and leading Indonesian policy field. Indeed, following Harmsen, it is Bologna’s “soft law” that has given it such persuasive power and reach:

Insofar as the Bologna process has been successful in creating a common European ‘language’ of higher education policy, with widely shared points of comparison, it has done so through the fostering of consensual dialogue, where no threat of imposed solutions…hangs in the balance. It is this absence of compulsion that has opened possibilities for policy learning, which have been significantly—if unevenly-seized. (Harmsen, 2015; 786)

Second, the article discusses how that European experts, by engaging directly with Indonesian actors, are made aware of opportunities to provide technical expertise in regional harmonisation and acquire a reach in ASEAN, far wider than the original bilateral relationship. Thus, the European Commission’s models and frameworks come to be used as a model in a world region, a region in which Indonesia aspires to play a leading role. The shifting geopolitical terrain in the global North, the economic rise of Southeast Asia and the widening influence and power of China (see European Commission, 1994, 1) have led European experts to look further afield for their new markets and alliances: Indonesia and ASEAN are the new frontier of the HE quality assurance expert dream. Of course, the benefits are mutual: not only does the European Commission acquire access to a regional education market, but Indonesia itself can present the country as having a globally benchmarked quality assurance system while assuming a leadership role in ASEAN’s quality assurance network.

Finally, the article builds upon and extends the emergent body of the literature that discusses the enhanced agency and ambition of countries of the Global South to actively seek and prioritise national sovereignty and independence, rather than ascribe to old mantra such as “West versus the Rest”. This is, as we will argue, the shifting geopolitics of expert knowledge production and use: on the one hand, under the influence of the Sustainable Development Goals, global governance has been characterised by a much more open, flat and participatory approach where older IOs’ zones of influence appear to demise. Indeed, within this political context, and the calls for decoloniality especially of those countries that until the second half of the twentieth century were colonised (such as Indonesia, though ironically, by European colonisers), the Global South not only has a say but is a key actor in decision-making: our research shows that Indonesian experts and policy actors have the confidence, ambition and capacity to play this role not only domestically but in ASEAN and globally. On the other hand, our argument acknowledges the dangers of applying a linear frame on the empirical findings that would merely see this either as a top-down or bottom-up policy: as the paper unfolds, we show the complex and multifaceted relationships and the distribution of power, ideas and interests among a range of actors and organisations, calling for a nuanced and careful analytical approach.

In terms of outlining how we develop our analysis, the article begins by engaging with concepts that emerge from a Science and Technology Studies’ theorisation of knowledge and expertise, and the sociology of quantification. We will move on to discuss our methods: these are primarily qualitative and have relied on text analysis as well as interviews with expert actors both in Indonesia and in Europe. The article will then proceed to offer some context for the analysis, followed by the presentation of the empirical data. The article will proceed with a discussion of our main findings and some concluding thoughts about the growing significance of global geopolitical shifts in the use and production of expertise.

## Theoretical and Methodological Resources: From “Governing by Numbers” to Epistemic Infrastructures of Measurement

Numbers proliferate in global governance: by creating comparable cross-border knowledge, they incite different institutional and political responses (Espeland & Saunder, 2007; Espeland & Stevens, 2008; Desrosières, 1998; Cussó & Piguet, 2023). A common reason for countries to engage with international actors is the need to be seen as working on challenges above and beyond their domestic politics. Often, getting the stamp of approval through alignment with international monitoring agendas legitimates local decision-making through processes of externalisation, i.e. seeking to find “approval” of national/local policy reforms from external actors that are considered expert, or lead on examples of best practice.

Recently, there is a growing recognition that the power and predominance of quantification has gone beyond the depoliticisation of political issues and has instead become a “logic” (Chun and Saunder, 2022) or a “culture” (Merry, 2016; Mennicken & Espeland, 2019). As such, quantification becomes more than a process of depoliticising policy problems: instead, it is a significant carrier of broader political, social, cultural and institutional orders.

This article follows this line of inquiry by exploring the processes and practices of quantification through the analytical lens of “epistemic infrastructures” (Bandola-Gill et al., 2022, 2024; Grek, 2022). Infrastructures as a social theory concept emerged for the first time in science and technology studies (STS) (Bowker, 1995; Star & Ruhleder, 1996). It was used to denote the mix of materials, practices and meanings that interconnect different knowledge structures, generating diverse impacts and changing social relations. Building on our extensive analysis of the term elsewhere (Tichenor et al. 2022; Grek et al., 2023), this article uses the notion of epistemic infrastructure of measurement as an interplay of three levels: the *materialities* of measurement; *interlinkages* between actors and measures; and finally, the development of *new paradigms* of performing education governance. As we will see, this analytical lens lends to theorising quantification as a meta-level phenomenon that has a governing function not only through its explicit political effects, but also—and crucially—through creating structures, connections and interdependencies, thus allowing for new governing spaces to emerge. This, in many ways, is the practice of “soft expertise”: not technical only, neither merely political, but both—creating new governing spaces that delineate the central idea and project, leaving enough room for local adaptations, translations and beginnings.

Thus, central to this article is the theorisation of quality assurance processes as an *epistemic infrastructure*: as we will show, the article documents the end of Indonesia’s heavy reliance on World Bank’s expert knowledge production as the top-down numerical steering of policy action, towards a new kind of soft expertise that the “European way” appears to offer: though still international and thus externally legitimated and “golden standard”, we will show how the epistemic infrastructure of the European quality assurance system has become the crucial site for a geopolitical shift in how Indonesia views and uses expertise; here, Indonesia serves as an example of broader shifts in the geopolitics of expert knowledge production, as we will discuss in the final section of this article. As European actors offer a measurement space that is as technical as it is deliberative and malleable, expert work does not merely count; it represents the key venue of policy deliberation, contestation and consensus.

Methodologically, the article uses qualitative empirical data derived from two research projects: first, we use interview data from 17 interviews with diverse European actors, from the European Commission’s Directorate General Education and Culture (DG EAC) officers, responsible for Higher Education, who discuss the perspective of DG EAC in relation to developing HE and the European quality assurance of universities with a global rather than merely European policy focus; the interview data are triangulated with discourse analysis of a key policy document which proclaims European universities as beacons of European values and culture in other world regions; this, in other words, is the “global Europe” that will—like it did in centuries past—emerge from the knowledge and influence of European universities. The data for this part of the analysis originates from the European Research Council project “International Organisations and the Rise of a Global Metrological Field” (METRO, 2017–2022). Second, the Indonesian case analysis is supported by data from a doctoral project, entitled “Expert Knowledge Flows for Higher Education Policy Making in Indonesia”, which examines the expert knowledge that supports the development of higher education policy, qualifications frameworks and quality assurance institutions for higher education in ASEAN and Indonesia. This research draws data from 35 interviews overall: 17 with Indonesian experts and policy makers, to provide the perspective of Indonesia’s ministries in selecting expertise and how this has changed over time, and 18 with experts from external agencies who have worked in Indonesia with Indonesian counterparts, to explore their understanding of and ambition for these partnerships. Interview data here are triangulated with and supported by documents from Indonesia’s government, the World Bank, the European Commission’s DG EAC and reports and documents emerging as part of technical assistance done in support of Indonesian and ASEAN higher education.

## Contextualising Europe, Indonesia and the geopolitics of global HE

Since the 1970s, within the European borders, there has been a systematic effort to create a minimum set of common objectives for European education systems. Although this lack of legislative competency of the EU in education is usually discussed as a challenge for governing European education, this section will contextualise the ways in which governing by coordination (or soft power, as described by Nye, 1990) has become the key vehicle via which European HE governance has become an influential global actor. This section also provides context to Indonesia’s HE system and its development of a HE quality assurance and accreditation system (BAN-PT) in the mid-1990s, which it has increasingly used to gain international visibility and access to global practices, such as through support from the EU (G BAN-PT). In doing so, this section gives a contextual overview of how European actors developed certain vehicles of coordination and then proceeds to explore the contexts in which these were taken up and adapted in ASEAN and Indonesia.

### The Central Role of Higher Education in the Making of the European Education Area

Universities in Europe have had a long history of being the beacons of European values, uniting European students, scholars and intellectuals for centuries. As discussed more extensively elsewhere (Lawn & Grek, 2012; Grek, 2008), the European political and economic project has always been fuelled by education and culture; the latter would help shape the agenda of a common people, and ultimately the “us and them” that distinguishes Europeanisation as the more benevolent and progressive form of globalisation—a process that began in the 1980s and continues apace:

Europeanisation found in the fields of education and culture some of the most influential carriers of a common European consciousness…’European citizenship’ would become the new unifying concept for the peoples of Europe, who, apart from being nationals, were now part of this sui generis project—the fabrication of European ‘culture’, or the European ‘civilisation’, as the French preferred. (Grek, 2008; 210-11)

More so than any other education levels and sectors, European higher education has always been seen as best placed to promote a degree of convergence around common frameworks and practices: from the success of the Erasmus project^2^ to the establishment of the European Universities policy initiative,^3^ higher education in Europe has always carried the promise and often the reality of creating Europe as a common, shared cultural space for young Europeans to enjoy (Lawn & Grek, 2012). The key role of European higher education in promoting mobility and quality to the project of Europeanisation can be exemplified in communication of September 2020 setting out the establishment of an “enabling framework” consisting of an embryonic form of governance body for the European Education Area:

**Figure:**
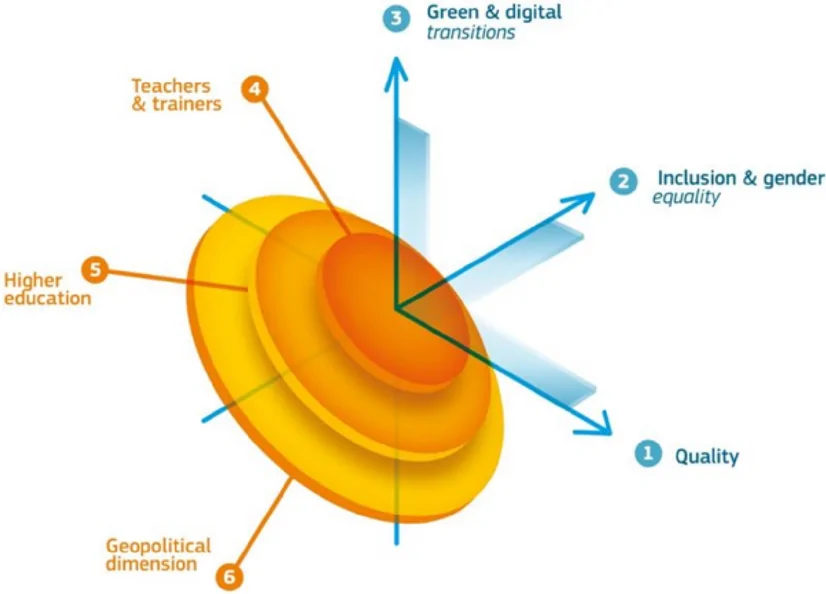

As the diagram shows, the dimensions of Higher Education, Geopolitics and Quality are key to this governing agenda, the materiality of which is manifest in the numerous policy documents outlining its key features, such as mobility, automatic recognition of diplomas, language learning, focus on culture, digitalisation, gender equity, green recovery and others. This is no coincidence since many of these elements of the European Education Area had also been key pillars of the development of the European Higher Education Area (EHEA). Further, the second level of the epistemic infrastructure of quality assurance has been key, exemplified through the interlinkages of two agencies as the key enablers of HE convergence: these are ENQA and EQAR. Indeed, one of the first organisations to emerge in connection with the initial Bologna developments was the European Association for Quality Assurance in Higher Education (ENQA). ENQA oversees the underlying infrastructure of QA in Europe (Ala‐Vähälä & Saarinen, 2009); as a result, its membership consists of European quality assurance agencies (QAAs). Given its central role, the Bologna Ministerial Communique called on ENQA in 2003 alongside other actors (the European Students’ Union, the European Universities Association and the European Association of Institutions in Higher Education) to develop an agreed set of standards, procedures and guidelines on QA (E4, 2011), the so-called European Standards and Guidelines (ESG).

ESG required the compliance of European universities by outlining a set of fairly broad and basic requirements. Enforced by the Bologna Process members, these standards and guidelines became key in defining higher education “quality”. Above all, a key characteristic of their value as material inscriptions is ESGs’ open and ambiguous character (Gornitzka & Stensaker, 2014; Brogger & Madsen, 2022). According to interview data from the METRO project, this was a way of resolving the age-old Europeanisation conundrum of trying to create some level of harmonisation of HE practices, while respecting the diversity of European higher education systems. The ESG suggested that although they aim at the “establishment of a widely shared set of underpinning values, expectations and good practice in relation to quality and its assurance”, plurality is “generally acknowledged as being one of the glories of Europe” and correspondingly “sets its face against a narrow, prescriptive and highly formulated approach to standards” (ENQA, 2005, p.10). As Harmsen supports (2015), keeping a studied ambiguity in the formulation of the ESG likely serves as a means of ensuring its acceptability to a wider range of European states and education systems. Rather than a strict standardisation, the ESG set out broad enough rules within which there is still scope for local translations and adaptations. Following the frame of epistemic infrastructures, the materiality of the ESG as a frame acts as networked tracks onto which many different kinds of national quality assurance frameworks could fit. Perhaps because of this breadth and ambiguity, it has been argued that the ESG has been one of the most successful harmonising elements of the Bologna Process (Bergan, 2019) and, as discussed by interviewees below, one of the main vehicles that European HE quality assurance could be exported to other world regions and systems.

This section focused on the context of the dominance of the quality assurance epistemic infrastructure, through a focus on the materialities (the ESG) and the interdependencies of the numerous European actors in this field. The next section turns to an overview of the Indonesian HE context post-independence to the present time.

### From Colonisation to Independence: Indonesia’s Aspiration to be an Equal Partner and Region Leader

Indonesia’s size, political history and complex alliances make it a geopolitically influential nation, rich in a diverse set of viewpoints and traditions. Indonesia is the world’s 4th most populous nation (267.7 million), with over 300 ethnic groups; it is the most populous Muslim nation and largest Muslim democracy. Around 43% of Indonesia’s population is under 25 years old, and its education system the third largest in Asia and the fourth largest in the world (Moeliodihardjo et al., 2013; OECD/ADB, 2015).

Indonesia’s HE system, governed primarily by the Ministry of Higher Education, Science and Technology and the Ministry of Religious Affairs (MoRA), developed from a diverse set of traditions including Islam and Islamic education as well as the education institutions established by the Dutch colonial government at the end of the eighteenth century (Buchori & Malik, 2004). Today, higher education in Indonesia is spread across public and private institutions and it is divided into institutions that are religious or secular as well as those that are vocational or academic. Higher education institutions (HEI)s are further divided by institutional type and include universities, institutes, colleges, polytechnics and academies (Logli, 2016). In public HEIs, staff—administrative and teaching—are civil servants appointed and managed by the National Civil Service Agency (BKN), whereas private institutions are mostly run by *Yayasan* (foundations) (Welch & Aziz, 2024). Private HEIs have increased in number from around 400 in 1980 (World Bank, 1980) to over 3000 in 2020 (Directorate General Higher Education, 2020) comprising 96% of all HEIs and 58% of enrolments (Welch & Aziz, 2024). The proliferation of private higher education has been the result of Indonesia’s greater reliance on the private sector to meet the growing demand arising from Indonesia’s young population, rising aspirations and growing middle class (Welch & Aziz, 2024). Despite an increase in general enrolment rates (from 11.5% in 1996 to 32% in 2016) (Welch & Aziz, 2024; UNICEF, 2018), there are disparities that correspond to income and geography. For example, in 2018, 2.5% of enrolments came from the poorest segments of society, while 65% came from the wealthiest segments (UNICEF, 2018) with an overrepresentation of students from urban centres and provinces like Jakarta, Yogyakarta and Papua Barat (UNICEF, 2018). HEIs are unevenly dispersed geographically with a higher concentration in Java and Sumatra and lower concentrations in Maluku, Papua and Tengarra (Moeliodihardjo, 2014). Improving the quality of higher education continues to be a priority area for the Indonesian government. Like other ASEAN member states, Indonesia aims to develop a “top tier” university (Welch, 2014, 148) and to participate in regional knowledge networks, which rely on higher education institutions. However, in the “overall world rank” of knowledge economy profiles, Indonesia ranks only 108, as contrasted with neighbours such as Australia, in 9th place, and Thailand, in 66th. Indonesia’s most recent strategic plan shows that it aims to grow its economy by 8% each year and it has identified obstacles such as a “gap in human capital” that needs to be addressed through higher education (see New Zealand Foreign Affairs and Trade, 2025).

The development of Indonesia’s higher education system was shaped by its colonial history and its subsequent participation in international development funding and technical assistance. Colonised by the Dutch from the seventeenth century and then by Japan from 1942 to 1945, Indonesia gained independence in 1945. After independence, Indonesia’s “free and active” foreign policy guided its approach to working with international organisations and other bilateral partners: Indonesia remained “free” from aligning with America or Russia in the cold war, and it remained “active” in its participation in world affairs, especially in “relaxing tension generated by two blocs” (Hatta, 1953, 444). President Sukarno later adopted a more confrontational foreign policy, “Konfrontasi”, that opposed the colonialism and exploitation inflicted by the “old established forces” of former colonisers (Agung, 1973, 314). Sukarno planned the 1955 Bandung Conference to unite African and Asian countries as a neutral front in the context of the Cold War (Van Reybrouck, 2020), and he withdrew Indonesia from the World Bank and the United Nations in 1965 (WB press release, 1965). Although Japan was also a former coloniser, it took a conciliatory role in the cold war and acted as a “bridge” between the “First world and Third World” (Garbagni, 2023, 392). Indonesia had normalised its diplomatic relations with Japan in the late 1950s (Agung, 1973, 266), and Japan proceeded to become the largest bilateral donor in Indonesian higher education over the next 60 years. Japan continues to influence Indonesian HE through providing facility improvements (since the 1970s) (JICA, 2009), academic exchange programmes (see Mubah, 2019) and bolstering university research capacity through various projects, which it has done since the 1990s (JICA, 2009; Ministry of Foreign Affairs of Japan, 2023).

After Sukarno’s fall from power, Soeharto assumed power and Indonesia re-adopted an “independent and active” foreign policy. At this time, a strong “anti-communist” stance was also espoused in response to China’s alignment with the Indonesian communist party in the 1965 coup (Mackie & MacIntyre, 1994). During Soeharto’s dictatorship, Indonesia rejoined the World Bank and ushered flows of foreign capital and aid, targeted at stabilising the country economically (Rosser, 2019). Soeharto’s dictatorship brought strong economic and educational growth, partly due to the oil booms of the 1970s. In the 1970s, the World Bank also started supporting Indonesian HE with loans and technical assistance, initially to provide manpower planning support for economic development (World Bank, 1975) and later, in the 1990s, with a focus on improving HE quality (see World Bank, 1991). From the late 1990s, the Bank supported the development of Indonesia’s National Quality Assurance and Accreditation Board of Higher Education (BAN-PT) as part of Indonesia’s New Paradigm reforms (World Bank, 1996). The World Bank’s support to BAN-PT continued after the 1998 Asian Financial Crisis (AFC) when Indonesia secured a rescue package with the IMF while undergoing democratic transition and political decentralisation (see Robinson & Rosser, 1998). For example, in 2005, the World Bank’s (2005), 50 million USD project, “Managing Higher Education for Relevance and Efficiency Project” (MHERE) was part of the technical support that bolstered BAN-PT’s capacity and supported the implementation of Indonesia’s Higher Education Long Term Strategy (HELTS).

Through the twenty-first century, as Indonesia transitioned to a democracy, scholars broadly note two shifts in its approach to engaging with bilateral and multilateral partners. First, Indonesia is claimed to have “recalibrated” its “non-aligned” stance into a “multi-aligned” approach. By this multi-aligned, or “pragmatic”, approach, Indonesia actively forms alliances with all the “world’s economic giants” (Wicaksana, 2022, 644), notably the USA, China, Russia, Japan and India, while also resisting taking sides in “bloc politics” between China and the USA (Gill, 2023). Second, Indonesia has continued to rebuild relations with China (Anwar, 2010, 349) despite lingering anti-Chinese sentiments that have erupted during times of economic difficulties. One example of this is the 1998 riots following the AFC, during which violence was aimed at ethnic Chinese (Zha, 2000). While remaining cautious of an assertive China in the region, notably in the South China Sea maritime disputes (see Dipua et al., 2020), Indonesia has embraced China’s technical assistance in higher education. China provides assistance through its Belt and Road Initiative (as mentioned), through student exchange programmes and through university partnerships (Oratmangun, 2022). In 2025, Indonesia confirmed its intention to bolster cooperation with China in higher education, research and technological innovation (Antara, 2025).

In the Southeast Asia region, Indonesia had its own imperial ambitions during Sukarno’s presidency based on what Sukarno believed as Indonesia’s “historic manifest destiny” and the “imperial grandeur of ancient Indonesian states like Shrivijaya and Mojapahit” (Agung, 1973, 414). While not considered “imperial” today, many scholars claim that Indonesia still has ambitions to take a leadership role in the Association of Southeast Asian Nations (ASEAN), formed in 1967, under the norms and values of the “ASEAN way”. The “ASEAN way” stresses a code of “non-interference in each other’s internal affairs, decision making by consensus and a minimalist approach towards regional integration” (Anwar, 2010, 345). Indonesia supports the “ASEAN way” and is trying to portray itself as “a moderate, peaceful and reliable partner in the region and beyond” (Heiduk, 2016, 7). However, the “ASEAN way” is sometimes put to the test given the tensions, agitations and conflicts in and among ASEAN member states. These tensions are complex and rooted in various historical, political, economic and religious divides. For example, Indonesia, Malaysia and Brunei Darussalam are Muslim majority countries and have faced pressure to defend Muslim minority groups in other states such as during the Rohingya conflict in Myanmar (Wicaksana, 2022). Additionally, only Indonesia, Malaysia, Philippines and Singapore are considered democracies, despite democracy being a stated value of ASEAN, which has been stated to compromise the formation of a common political identity (see Rautakivi & Yolles, 2024). Further, ASEAN member countries have divergent (sometimes clashing) motivations for and preferred methods of regional integration. Thailand, Malaysia and Singapore tend to seek greater economic integration, while Indonesia tends to push security cooperation, while adopting a more protectionist approach in some areas of economic regional integration (Heiduk, 2016).

Although not part of ASEAN, Australia is a prominent regional partner and uses its aid (including education aid), of which Indonesia is one of the largest recipients, as an extension of its foreign policy (Welch 2014). Australian universities further host a significant number of Indonesian and ASEAN higher education students. While Australia values the growing source of revenue this provides, there is a conflicting desire to reduce the labour mobility resulting from international students, who formed over half of permanent resident applicants in the mid-2010s (Welch 2014). Australia is starting to participate more in regional forums such as the Southeast Asian Ministers of Education Organisation (SEAMEO) and is motivated to participate in regional knowledge networks. Just as this research was ending, Australia partnered with several Indonesian Ministries and agencies to form connections between Australian and Indonesian knowledge and innovation organisations (Australia Department of Foreign Affairs and Trade, n.d.).

In recent decades, Indonesia has positioned itself as a key global and regional player. In 2022, Indonesia hosted the G20 Summit which included an education working group led by Director Generals from Indonesia’s Ministry of Education and Culture. Commenting on the Summit in a 2024 speech, Indonesia’s Minister for Foreign Affairs, Retno Marsudi, remarked that it was “evident that Indonesia is a key player both regionally and globally, and NOT merely a spectator” (Marsudi, 2024). She continued by noting that:

not many countries are able to consistently play the bridge-building role well in the midst of very sharp differences in views between countries. Against all odds, Indonesia was actually able to revive the spirit of collaboration among G20 members and prove to the world that developing countries can indeed determine the course of negotiations (Marsudi, 2024).

Indonesia is also considered a “major force” in regional governance forums, and it actively participates in global and regional HE quality assurance networks. In 2023, Indonesia assumed the rotating Chairmanship of ASEAN, and it is a member of SEAMEO’s Regional Institute of Higher Education (RIHED) (Welch and Aziz, 2024, 378). Indonesia’s ministers have encouraged its QA experts to take leadership roles in wider regional and global QA networks such as the ASEAN Quality Assurance Network (AQAN), the International Network for Quality Assurance Agencies in Higher Education (INQAAHE) and the Association of Quality Assurance Agencies of the Islamic World (AQAAIW). In more recent years, Indonesia’s QA experts have been advisors in other regions and countries such as Mongolia, Sri Lanka, Jordan and Namibia as part of World Bank projects, Asian Development Bank projects and INQAAHE forums. Thus, as European actors provide more support to HE harmonisation in ASEAN, most recently through the 2015-2023 project EU Support to Higher Education in the ASEAN Region (EU SHARE), QA infrastructures serve as a network and forum where Indonesia’s experts seek not only access to global knowledge but also an avenue to widen their own influence. Through joining and forming networks with ENQA experts, Indonesian experts have extended their reach just as have European actors. In fact, it is the mutual benefit of this arrangement that following sections will show Indonesia’s experts to value.

In sum, a shift is emerging as Indonesia takes more charge of the terms of engagement of expert exchange, while also playing more of a lead role in providing expertise to other regions. The next empirical section returns to Europe: it focuses on the working ways of European quality assurance experts that arguably make them suitable to the Indonesian context.

## “Soft” Expertise and the Global Expansion of European HE

What are the qualities and aspirations of European HE policy makers and quality assurance experts that make them good collaborators and partners for their Indonesian counterparts? As multiple interviewees in the METRO study related, a common feature of European QA experts is that they share a background as academics who moved to the quality assurance field from previously having held university and policy leadership positions managing quality and accreditation. This double—and often triple—identity is often seen as generating trust, since it signifies their work on academic quality as an adherence to the fundamental principles of academic excellence and rigour, rather than merely as a technical, or technocratic matter. Many European actors that work in quality assurance organisations or have been closely involved with the Bologna Process would often signal their academic affiliation as a sign of moral and epistemic integrity and experience (“I’m also a professor in higher education in the field of chemical engineering, and work also in the ministry”, CG ENQA). More important, such multiple affiliations signal their deep knowledge of the complexity of governing diverse academic institutions, despite their belonging to a larger European family. In a similar manner, through our work with Indonesian experts, we found most of them to also be lecturers, professors and/or deans of universities in Indonesia, sometimes holding these roles while also holding a role in Indonesia’s Ministry of Education. One Indonesian interviewee echoed that this double affiliation indicated a deep knowledge of what was good for the sector and country:

[W]hen I make policy in the Ministry, then I can check whether or not the policy is… you know… executed in the classroom… I can make a proper policy that works for people because I know the situation on campus. Many of the, sorry to say, bureaucrats and the officials in the ministry, when they make decision, most of them don’t know what is going on in the campus (AC MOEC)

The intersection of professional and academic career trajectories might be one of the reasons that experts in the field appear to form a consensus about the benefits of the interdependencies of the quality assurance infrastructure. In addition, the “softer” modus operandi of European QA allows highly adaptable processes and practices, respecting both the context and diversity of European HE systems, as well as the rule of subsidiarity that has shaped European education.

Above all, and as explained earlier in this text, although European HE has always driven the convergence agenda in the European education policy space, respecting national differences and countries’ sovereignty in making decisions about educational matters remains intact. Thus, although alignment with quality assurance standards is achieved through agreeing on a number of fundamental and broadly defined parameters, plenty of space for divergence is also allowed. As discussed in a previous section, this soft governance of quality in European HE is primarily facilitated by the material infrastructure of the ESG, which allows both alignment (“go in the same direction”) and divergence (“vastly different”). According to a METRO interviewee:

My hope would be that for the European universities, we can try to go in the same direction as we have already been trying to go with joint programmes and say OK for now we have this ESG, which are a set of European standards, but they are more like a framework standards. So we still have very, very vastly different quality assurance systems in Europe, in each country. They all have their national criteria, they all have their national approaches that are based on the ESG but that still differ a lot. (CT int.)

Another METRO interviewee stated:

One of the reasons for which the ESGs have been so successful was the way in which they were developed. So, prescriptive enough in order to induce the change needed, but also general enough to have so many countries being able to work with it, and then becoming compliant (CG int.)

A DG EAC actor, interviewed for the METRO project and working in the field of HE QA, also stressed the success of following a collaborative, “cooperative arrangement” that is voluntary as a key ingredient of the European HE quality assurance success, and as we will see, its export, too. Above all, the success of the instrument comes down to its ability to create alliances and find common ground for the wide range of different stakeholders that play their role in quality assurance:

(We have) working groups and we have EU Member States, we also have EEA countries, we have candidate countries and then we have international organisations, like UNESCO, Council of Europe, the OECD, we have stakeholder organisations, like ESU, EUA, EURASHE, EQAR…So, I think that side, it actually works very well, I think given the longstanding cooperation and given the fact that our cooperation is voluntary, that we value the input we get from those groups…it’s a very, very cooperative arrangement (EC6).

Indeed, rather than pre-specified and led from “above”, the epistemic infrastructure of European QA grew slowly and “organically” (Dakowska, 2020; Fumasoli et al., 2018) through different opportunities and possibilities, as they emerged over time and space over the last 20 years. As we will see in the next section, similar to the European context, the epistemic infrastructure of quality assurance in Indonesia grew, linking European *and* Indonesian ideas, interests, knowledge and practices over time and space in a new infrastructure, away from the European “home”. Within this new context, the role of “soft expertise” was to facilitate the building-up of the epistemic infrastructure, as a mechanism to produce new knowledge and practice and as a common space of socialisation and culture. Thus, by soft expertise we refer to the process of bringing together a divergence of actors and interests and making them align around focal values and ambitions. In other words, soft expertise is the function of brokerage and mediation (Grek, 2022; Bandola-Gill et al., 2022) that glues materialities and actors together into a new governing paradigm, the third “level” of the infrastructure: in the Indonesian case, this has meant the end of an era of dependency on the WB, towards a new condition that is far more self-directed and horizontal, and that has been mutually beneficial to both European and Indonesian actors in their efforts to play a bigger role in the ASEAN region.

Such expansion for European HE is key: In a world shaken by the rise of—overt or covert—authoritarian regimes, carrying the purported EU values of democracy, equality, peace and rule of law to other world regions becomes a prime geopolitical aim, especially at times when major global powers retreat to more protectionist agendas and the EU itself is stricken by the rise of populism and hard- right parties that question the very purpose and value of the EU project itself. In addition, if geopolitical influence is about distributing the global balance of power, such “soft”, cultural influences become particularly important, alongside of course the economic expansion through the harmonisation of the different HE markets that will allow future mobility, scientific exchange and growth.

Indeed, in expanding into Southeast Asia, European actors were seen to collaborate regularly with actors in ASEAN: this was not seen as an easy task, despite the lengthy experience that European actors had with coping with divergent interests, traditions and practices within the European context and despite having some foothold in Indonesia already through ENQA’s involvement in QA capacity building. Entering a new territory in supporting ASEAN higher education meant careful stepping into the region, ensuring that good working relations are established not only with national actors, but also with regional agencies such as SEAMEO and other IOs that had already been active in regionalisation in the Asia Pacific.

According to a DG EAC official,

One thing that I saw in the data from last year was that there were more cross-border external quality assurance activities carried out outside the European Higher Education Area for the first time than within the European Higher Education Area. So there is an increasing interest from countries that are either bordering the European Higher Education Area, from Latin America for instance, to get this accreditation from European agencies (EC9)

In particular, collaborations with other IOs, such as the OECD, facilitate the data interfacing of the European quality infrastructure with, and potential extension into, other world regions. The European Commission’s DG EAC has explicitly stated its aspiration for European Higher Education to become a global actor:

We have quite an important focus and interest and activities developed from beyond Europe. Of course the interest for exchanging experience, because there are also other networks of quality assurance agencies for all over the globe, African, Asian, United States, so the collaboration with those networks is important. So what we try to do is to learn from each other, but also our objective is to promote the European standards and guidelines, because of course we believe they are good. Well, it’s good to go beyond Europe with that (CG int.)

Thus, and to conclude, the epistemic infrastructuring of quality assurance in Europe has allowed its expansion to new geographies and regions. The travelling of European expertise was successful not only due to its technical capacities and skills, but crucially thanks to its (geo)political sensitivity that allowed it to understand local idiosyncrasies, create space for some harmonisation *and* freedom and thus increase its clout and influence, as the European HE dream has always been:

I think we have been reaching that point, we have been getting to that point and it’s funny you mention it, because developing this European brand of Higher Education was actually the main purpose of the Bologna Process in a way, increasing the attractiveness of the European Higher Education Area. So seeing that this is a big interest for countries outside EHEA I would say we have been doing a good job. We have got there eventually, 20-something years later! (EC3)

The next section will discuss the ways that European actors managed these processes and negotiations; it will show the role of soft expertise and the reasons that it became a preferred method versus the previous experience Indonesians had with the World Bank; and finally the section will lead into the discussion of the empirical data, focusing on the increasing influence of geopolitics in the production of expertise in the twenty-first century.

## European Actors in ASEAN and Indonesia

Europe’s influence in Indonesia, through the ASEAN Secretariat, provides a context to examine the role of soft expertise. European influence in ASEAN and Indonesia was enabled partly by Europe’s brand of HE—having the distinctive quality assurance and qualifications frameworks that facilitated regionalisation—made available at the right time, when there was “political will at the regional level in Southeast Asia to foster harmonisation” (BE SHARE) in HE, which one European expert perceived to be a “less higher stakes form of collaboration across the region states” (CE int.). Representatives of the EU delegation to Indonesia noted an increasing “desire for both the EU and ASEAN, the ASEAN secretariat, to work more closely together” (CE int. and DN int.), and the 2015 establishment of the EU delegation to ASEAN provided an institutional space for these collaborations. Thus, Europe “provided expertise, knowledge and resources to make it [regional harmonisation] happen” (EN int.) through a 15-million-euro grant-funded project called the EU Support to Higher Education in the ASEAN Region (SHARE) Programme, which funded workshops and training in the areas of QA and qualification frameworks, policy dialogues and intra-ASEAN and EU-ASEAN scholarships. The EU SHARE project (2015–2023) worked through the ASEAN Secretariat—located in Jakarta—to:

support the harmonisation of higher education in the ASEAN region by building on relevant experiences and expertise from the European Bologna Process, the ERASMUS mobility scheme and the creation of the European Higher Education Area (EU SHARE, 2019).

Europe’s brand of HE became a central point of reflection in creating the “ASEAN HE space, which is inspired by the Bologna Area” (FE int.). As one European expert from the project stated: it was “by design and not by coincidence that there are similarities between what’s happened in ASEAN and what happened in Europe” (BE int.). Indeed, expert work blended the political task of region building with technical tasks in developing regional frameworks and instruments with reference to similar ones in Europe. One example was ASEAN’s regional QA framework (AQAF). According to an interviewee from Indonesia’s QA system, “the AQAF, the framework, was very much—it’s not copied but it’s—when we developed that, we have a very good support from ENQA” (GT int.). European experts were eager to bring their experiences into ASEAN, and one interviewee from the EC stated that

we’ve been through our Bologna Process. We’ve set up these systems for credit transfer, for recognition… all these things we think work. And so we were quite keen that, you know, ASEAN should try out the same sort of approach (HC int.).

Soft expertise became the vehicle for doing so, bridging boundaries through dialogue and developing common frameworks and instruments while allowing room for local divergence. European experts did not perceive their work as a “copy paste” job (IA int., CS int., LE int., JA int.), but rather as finding ways of better understanding systems to accommodate differences and find linkages. As an European QA expert stated, the work was “to kind of understand, ok, you have this thing, and we have this thing. How do they connect? Where are they different and how can we make them more interoperable” (IA int.).

Thus, through various networks and dialogue forums—as part of EU SHARE and the EHEA Coordination Group on Global Policy Dialogue, which started engaging with other regions in 2018—European actors, Indonesian experts and ASEAN representatives played a brokerage role to bring together the actors and materialities for harmonising higher education systems in ASEAN with reference to the Bologna Process. The success of these expert brokers did not of course come out of nowhere: the Bologna Process itself, through its “External Dimensions” Working Group has pushed and has largely been successful in pursuing extra-European cooperation, in regions of the world as far as Africa, in Central Asia, and with influence in countries such as Canada, New Zealand and Israel (Zmas, 2015; Moscovitz & Zahavi, 2018; Alemu, 2018; Asderaki, 2018; Klemenčič, 2019); in that sense, although a European process, the Bologna Process has become a global force, and thus, translating its key principles in Indonesia should not come as a surprise. Nonetheless, even in occasions when priorities did not align between European and ASEAN actors, this was not seen as a challenge for QA experts who were flexible in facilitating local inscriptions of quality and divergent ways of operationalising frameworks and processes at regional and national levels (GT int., LE int.). Similar to the ESG, Indonesian QA experts noted the effectiveness of making a “very generic type of [QA] instrument… [while] [a]t the institution level, of course, we recognise such mission differentiation” (GT int.), and this was noted to accommodate a diverse set of HE institutions in ASEAN and within Indonesia (AC int.). Local adaptations were also observed in developing the ASEAN qualification reference framework (AQRF), to which Indonesia’s qualification framework expert claimed to add an “Asian vision, that we are together”, which was considered a “kind of philosophy” that appealed to stakeholders in “Africa, India and Pakistan” (AD int.). As such, Indonesia’s experts were able to add their own expertise to these infrastructures in a way that enabled them to extend their own visibility and reach into other regions, and “international visibility” was stated as an aim for Indonesia’s QA experts as well (GT int.).

For most of the Indonesian experts interviewed, a key criterion for bridging boundaries was to establish terms of cooperation that built upon infrastructures in a way that was mutually beneficial, and this principle was emphasised by one civil servant from Indonesia’s Ministry of Education and Culture:

Collaboration is important, but collaboration is important with the mutual uh… benefit scheme. Most of the benefit from the collaboration is one give and the other… they cannot give back in the same level yeah… mutual benefit (AC int.).

An example of this is evident in that while facilitating student mobility was stated as the overall aim of EU support to ASEAN HE (see EU SHARE, 2018), it was not a priority shared by Indonesian experts. Indonesian interviewees “question[ed] whether the government can really sustain the funding for that… also from the academic standpoint, we start questioning whether mobility should be reciprocal” (GT int.). Indeed, European experts acknowledged that “their priorities are not ours” and “mobility is not the universal experience” (CS int.), and they bridged gaps such as these by identifying areas of collaboration that could be mutually agreed. In this case, as stated by an European higher education expert, collaboration focused on technical work in developing and linking systems: “practical steps would be reciprocal referencing of our qualification frameworks, or reciprocal referencing of our credit systems… these sorts of things, for me, are starting points” (CS int.). Even Indonesian experts who were sceptical about student mobility acknowledged the work done to develop and harmonise QA systems and qualification frameworks as a “main achievement” of the project (GT int.).

In this work, alliances were complex and required careful negotiation to allay tensions and accommodate preferences. Some Indonesian experts perceived European experts as trusted mediators who were able to bridge differences between ASEAN member states, claiming there to be more “challenge working with our own neighbours” than with European experts. While there was “necessity to frankly tell the challenges or difficulties to experts from Europe because it’s too far away”, they would “hesitate to become open with our own neighbours… that’s natural” (MC int.). Indeed, one expert from the EU SHARE project contrasted the external image of “ASEAN, we are one” with the internal lack of trust when it came to quality assurance systems, explaining that “if you get them on their own, they don’t trust each other” (NE int.). Thus, European experts perceived themselves to act, in the words of one interviewee, as “honest broker[s]” (CE int.) in bringing together various regional actors. Yet, as a bloc, ASEAN firmly rejected parts of the Bologna process, and work was adapted to accommodate these objections. For example, while Europe encourages “strong student representation at our universities”, one interviewee from SHARE stated that “they [ASEAN members] were quite adamant that this is not something they would want to pursue to the extent that it is done in the EU” (FE int.). Additionally, this interviewee stated that ASEAN member countries maintained a system of “voluntary compliance” with the conditions of regional quality associations rather than implementing the strict reviews that apply to Europe. The sentiment of ASEAN countries was perceived to be: “we want to comply to this but its voluntary compliance and that became kind of the term here” (FE int.).

Finally, while formally partnering with the ASEAN Secretariat and supporting the build-up of regional frameworks, EU experts also built connections directly at the national level in Indonesia through consultations and “capacity building” for QA and in referencing national frameworks to newly developed regional frameworks (BE int. and GT int.). As noted, by the time of EU support to ASEAN HE, Indonesia had already participated in growing global QA networks. Further, Indonesian and European experts claimed to already have “very well-established working relations” through the ASEAN-QA programme (BE int.), which built national capacity in Indonesia’s internal and external quality assurance evaluations (GT int. and FE int.). ASEAN-QA was jointly run by stakeholder organisations such as AQAN and ENQA, among others, and it had promoted the relationships and processes that the formal EU project built upon. The importance of good personal relationships was consistently stated by interviewees. For European experts, while the “entry gateway into these activities was [at first] these big networks”, now good relationships had become much more informal and direct, allowing ease of communication: “I would just call the director of the Indonesian quality assurance agency” (IA int.).

### Waning Support of the WB and other Actors in HE

The approach European actors took to use regional forums to move into the HE policy space in Indonesia came at a time when Indonesia’s key bilateral development partners were becoming less invested in HE. For example, Australia’s support through the Inovasi project, which runs from 2016 until at least 2027, was mentioned by Indonesian civil servants as a go-to source of expertise in education (RC int.). However, Australia’s Department of Foreign Affairs and Trade (DFAT), which manages the project, focused the work on primary education, having cut HE work due to “recognition through the PISA results… that student learning was so poor that the focus, or DFAT’s focus, would remain at that basic education level” (PT int.).

As noted, another key partner up to this point had been the World Bank (WB). Indonesian interviewees mention the Bank as a main source of expertise in establishing BAN-PT (GT int.) and a gateway to global knowledge. One Indonesian expert reflected on working with the Bank on projects in Indonesia in the 1990s and 2000s: “They bring people from all over the world, you know…[from] Latin America, from Washington, from anywhere in the world, to do the work. They usually pick up top-notch guys” (TC int.). While many interviewees from Indonesia’s HE system perceived the World Bank’s expertise from that era to have been valuable (AC int. and TC int.), reports and interviews also stated that Indonesia was not reliant on the loans the Bank provided for education (SC int.). Bank reports from the mid-1970s state the “growing capacity of the government to finance its own development projects independently” (World Bank, 1975, 23), and that “Bank financed projects represented only a small portion of the Government’s program. The bulk of funds came from the Government’s budget” (1991, viii). However, over the last decade, the Bank is noted by interviewees to be increasingly absent from higher education policy work and to have a difficult time receiving attention from Indonesia’s ministry of education (SC int.). Several reasons for this were noted by interviewees. One reason was stated to be public criticism levelled against Indonesia’s government for the decades of loans taken from the Bank (VS int. and WS int.), loans which had strict compliance and procurement processes. One interviewee commented on the compliance hurdles faced by the Ministry of Religious Affairs (MoRA) which had recently taken a WB education loan. The Bank was claimed to have “A lot of compliance that they are not ready for. And then they didn’t realise that… and… the Bank is… Bank is Bank. What is written is written” (TC int.).

The rigidity of WB processes is not a new complaint from Indonesia’s bureaucrats. In a 1991 Brookings institute interview with Saleh Afiff, chairman of Bappenas at the time of Soeharto’s dictatorship, Afiff calls WB processes a “theology”, adding that “[w]e are more flexible. I mean, you know, we don’t have that type of theology. We are more flexible…” (1991, 26). Thus, as formally stated in a 2015 independent review of the WB project MHERE, “the [Indonesian] government has decided to stop borrowing for education projects” (IEG, 2015, n.p.). The statement of an education expert working in the field is also telling in relaying the perception that the WB team working in Indonesia is not as strong as it once was, which was understood to indicate that Indonesia is not taking the same level of loans as the era when the Bank brought “top-notch” experts to the field:

I remember when… so when Nadiem first came in as education Minister I think there was kind of renewed optimism that being you know an American schooled, you know, American graduate, that he may be more open to um, World Bank funding… I mean their [WB] team here in education, yeah, they don’t have the same, I guess the strength of the team that they used to have and that probably reflects that they don’t have the loans, right, which allow them to bring global world class expertise to come [to Indonesia]-. (PT int.)

The rigidity of WB expertise was also perceived in the way the WB worked with Indonesian agencies, giving them less room to steer the agenda. One international development consultant noted that “there was some dissatisfaction with the World Bank” (WS int.), specifically on a programme where the Bank acted as the executing agency for an education development fund provided by an assortment of donors. Thus, as an offshoot programme emerged, the Bank was replaced by the Asian Development Bank, and a lead consultant on the programme recalled that “they’re [the WB] considered to be a know-it-all and uh.. rather dictatorial”. Continuing, the consultant stated that

the problem for the World Bank dated from the Soeharto era where World Bank had… run up a huge debt for Indonesia.. uh and had not addressed issues of corruption. So, after the reform and uh democratic rule they got a lot of criticism. But I don’t think this was such an issue at this stage. At this stage it was more that their experts were a bit, dictatorial. And the counterpart… wanted to have the project be much more led by the counterpart agencies, and not by the donor community (WS int.).

Indeed, interviewees from Indonesia’s civil service emphasised Indonesia’s ability and resolution to steer expert knowledge production—through using, limiting and directing it—according to its own agenda, stating:

[T]he Ministry has the capacity to be able to define our own goals and our own strategies and so we make use of whatever resources that we have including external experts, um, be it from the World Bank, from other foreign governments from ASEAN as we see fit… So, it’s us driving the agenda, uh, mostly. And…we are, like giving much more specific requests and instructions to those foreign agencies at the moment with regards to, this is what you should do. If you continue to do that, we won’t be um… giving any attention to whatever results that you come up with because this is our agenda (RC int.).

The rigid approach to loans and expertise can be contrasted to European Commission’s approach, as discussed in the previous section, which was open to a more flexible system of compliance and negotiated more nimbly to accommodate divergent priorities and values.

## Discussion

Building on a rich set of policy documents and interview data, and using the concept of the epistemic infrastructure, the article focused on an analysis of how European HE expert actors became the preferred advisers for Indonesia’s HE quality assurance system. The empirical evidence confirms the rich scholarship on the sociological analysis of quantification; the latter has seen the role of quality assurance data, and all the associated processes to collect, validate and harmonise them, as governing tools that steer not only policy problems and their solutions, but also their producers and receivers, and the relationships that bind them. As this article has showed, working towards the quality assurance of higher education institutions in Indonesia serves here not only for the apparent purpose of offering technocratic and rational solutions to improving HE governance and delivery, but also for the parallel aim of expanding the role and influence of European universities in other world regions and zones of influence. Given the role and identity of the EU as a political project, as well as the specific mission that has been assigned to European universities as custodians and carriers of European liberal values, the work of European experts in Indonesia has geopolitical value and significance; it thus allows us to examine the geopolitics of expert knowledge production as a space of interactions, possibilities and contradictions.

In particular, the article showed the ways in which the material inscription of the ESG as an open “framework” document guided the careful treading of European experts in establishing partnerships, offering solutions and allowing space for Indonesian actors to take initiative and ownership of the work. Such an approach was successful, since it represents a leap in the practice of transnational expert work: although prescriptive, it also appears as transparent, pluralistic, open and developmental—consensus-making is prioritised and consultation processes are required in order to understand and respect the local context. What we observe in this setting is an emergence of a specific form of expertise—one that goes beyond simply focusing on producing and disseminating knowledge. Instead, this work requires creating specific epistemic infrastructures in which knowledge is agreed upon and produced. This activity goes beyond simple acts of translation and adaptations to the local context: on the contrary, the interplay between European and Indonesian experts involves setting the conditions in which knowledge will be produced through setting standards, coordinating their application and finally, navigating the communication between diverse networks of actors. This makes the work of soft expertise not only one of a brokering connections (Meyer, 2010) but also one of an architect: this work would involve getting the policymakers involved in setting the priorities, choosing the dimensions and shaping the process. Coordination is complex, requiring bridging multiple boundaries at once—including boundaries between different institutional settings, epistemic boundaries between different forms of knowledge and differences in practices. Even though European experts saw their role as predominantly supporting quality assurance processes, soft expertise was more complex work than translation between knowledge and action. Instead, they have to navigate between different knowledge systems—from scientific, political, economic to local, indigenous, decolonial and practice-based. Further, in relation to the work of infrastructuring quality assurance, a particular boundary that was facilitated by European soft expertise was the practice one—including workshops, training sessions, online learning and handbooks aimed at capacity building. These strategies were not just aimed at creating specific knowledge repertoires for the stakeholders to use but they have a more important role in creating what Galison (1997) called the “rules of exchange”. These rules of exchange allow for coordinating and collaborating between different actors without necessarily requiring their consensus, thus allowing for some harmonisation of agendas while leaving enough space for local interpretations.

Nevertheless, these processes are not smooth and linear. As the empirical material shows, they involve allegiances and antagonisms among and between the actors involved, and increasingly so as new rivalling global sources of expertise become available to Indonesia. At the same time, there is a growing perception inside the country (but also outside) that Indonesia now has the internal capability to define and solve its own higher education problems. Indeed, in a changing, multipolar geopolitical landscape, expertise creates and sustains international partnerships, while also softly steering national measurement practices and policy priorities. With China investing in “connectivity” in Southeast Asia through developing regional higher education links—among other programmes of China’s Belt and Road initiative, European actors are competing for influence through establishing their models and frameworks for regional higher education harmonisation. There is competition in providing expertise to countries of the Global South, made apparent in the firm discretion Indonesian civil servants and experts have in their selection of the external sources of expertise they employ. Such discretion is applied not only in the experts they work with but also in the fields they use this expertise for: as shown in the analysis above, Indonesia has showed significant agency in restricting the evidence IOs supply for policy making to only topics that align with the priority areas and reforms Indonesia has determined for higher education. This stands in contrast to decades of working with the World Bank, where the WB would decide and design the studies to identify problems in Indonesian higher education and implement solutions. In this landscape, the soft expertise of European actors is befittingly agile, accommodating national and institutional values and standards while holding coherence with a few “minimum” criteria of quality assurance.

To conclude, the newly developed relationship in higher education between the European Commission’s DG EAC and Indonesia represents a clear case of the role and influence that expertise can have in shaping geopolitics, particularly in an era of increased calls for the global South to shape its own future, coupled with the retrenchment of the USA in respect to development aid and global influence. At the same time, as we discussed, despite the apparent soft expertise of the European actors and officials, their presence and work in Indonesia cannot be decoupled from the relatively recent history of European colonial rule. Our analysis calls for two ways of drawing from innovations made to STS analytical frameworks: first, by incorporating insights from postcolonial theories and, second, offering new challenges to theories of decoloniality.

First, as Cardwell et al. (2022) make clear, “some infrastructures are more powerful and entrenched than others”, and they indicate the obduracy—yet not immutability—of some ways of knowing as shaped by epistemic infrastructures in postcolonial contexts. Yet, these scholars also emphasise the importance of the way in which actors draw on epistemic infrastructures, and this is not fixed. Even with entrenched epistemic infrastructures, “if they are artfully drawn on and arranged, alternative ways of knowing are possible” (Cardwell et al., 2022; drawing from de Sousa Santos, 2016). Indeed, it is likely that this is a starting point given the difficulty of going against the grain of entrenched path dependencies and local cultures. Based on the empirical evidence of this case, future research might focus on how quality assurance infrastructures work in local universities in the Global South, and the extent to which they enable or marginalise alternative ways of knowing. Second, challenges to theories of decoloniality provide a vital starting point for understanding, due to the evident multidirectional, multilayered and multifaceted flows of power, that an analytical top-down frame would have been blind towards. For example, several strategies for control and power can be uncovered by analysing exchanges between China, Europe and ASEAN countries in competing for influence in ASEAN. Understanding the role of geopolitics of expertise—in shaping and steering these complex relationships—requires a decentred way of analysing de- or neo-coloniality: this would need to move away from simplistic “top-down” analyses, towards an understanding of the geopolitics of expertise as an intricate and continuously evolving epistemic infrastructure, where all actors (colonies and colonisers included) play their own part in governing higher education.

## Footnotes

1 In this paper, we follow the scholarly literature which identifies the European Union, and one of its core international administrations, the European Commission, as an intergovernmental organisation, and as such similar in function and role as other major IGOs, such as the NATO, OPEC, the OECD and the World Bank (Cini, 1996; Hooghe, 2001, 2005, 2012; Kassim, 2020). Indeed, some of these IGOs are global and not regional; our paper develops an argument about how the European Commission, a regional IGO, may lay claims towards becoming an IGO with global significance and power.

2 The Erasmus+ programme is a European Union initiative that supports education, training, youth and sport. It provides funding for individuals and organisations to engage in mobility, cooperation and capacity building in these fields.

3 According to the European Commission webpages, “The European Universities alliances are part of a flagship initiative of the European strategy for universities. They encompass 65 European Universities alliances with more than 570 higher education institutions of all types, from all across Europe” (For a rich analysis of the policy in relation to Europeanisation, see Brooks and Rensimer 2023).
